# Does sedation with AQUI-S^®^ mitigate transport stress and post transport mortality in ballan wrasse (*Labrus bergylta*e)?

**DOI:** 10.3389/fvets.2024.1347062

**Published:** 2024-01-15

**Authors:** Sara Calabrese, Thor Magne Jonassen, Endre Steigum, Helga Øen Åsnes, Albert Kjartan Dagbjartarson Imsland, Carolina Serra Saude, Truls Wergeland, Erik Höglund

**Affiliations:** ^1^Norwegian Institute for Water Research, Bergen, Norway; ^2^Akvaplan-niva AS, Tromsø, Norway; ^3^Department of Biological Sciences, University of Bergen, Bergen, Norway; ^4^MOWI ASA, Bergen, Norway; ^5^University of Agder, Kristiansand, Norway

**Keywords:** delayed mortality, sedation, transport, Cleanerfish, aquaculture, fish welfare

## Abstract

Ballan wrasse (*Labrus bergylta*) are commonly used as cleaner fish in salmon farms as a biological treatment to mitigate sea lice infestation. Improved welfare for cleaner fish both during production of these fish and when in sea-cages with salmon is crucial for the industry’s development. A common operational procedure in ballan wrasse production is transporting juveniles from one land-based farm to another for further on-growing. Episodes of increased mortality have been reported after such transportations. In this study, the relationship between transport stress and post-transport mortality at the on-growing facility was examined. It was also investigated if light sedation with AQUI-S^®^ can mitigate stress during transport. Stress was quantified by measuring cortisol release rate to the tank water during transport. This was investigated in 10 commercial live carrier truck transports (6 without AQUI-S^®^ sedation and 4 with sedation during loading and transport). The total time of transport varied between 12 and 21 h. In general, mortality was significantly higher (1.0 ± 0.6% day^−1^) the first five days post-transport compared to 15–20 days post transport (0.5% day^−1^). There was also a strong relationship between fish weight at transport and post-transport mortality, where higher mean weight at transport reduced mortality. In contrast to what was expected, AQUI-S^®^ treatment during transport procedures increased cortisol excretion rate, suggesting a stimulating effect of AQUI-S^®^ on the stress axis in ballan wrasse. Considering these results, the value of using AQUI-S^®^ to reduce stress during transport of juvenile ballan wrasse might be questioned. However, there was no relationship between cortisol release rate during transport and post-transport mortality. Furthermore, this study emphasizes that water cortisol measurements can be used as a none-invasive tool for monitoring stress and can be integrated into the welfare evaluation during commercial fish transports.

## 1 Introduction

Sea lice control continues to be the primary challenge for growth and sustainability of the Atlantic salmon farming industry in Norway and elsewhere (1–3). There is currently no effective vaccine against sea-lice. In addition, sea-lice show increased resistance to chemo therapeutics (2). Therefore, non-medicinal solutions for sea-lice control are the most common mitigation strategies. A biological strategy, using cleaner fish to delice salmon is now commonly used and both reduces the need for medicinal treatments and is less stressful for the farmed salmon compared to other delousing methods that require extensive handling (4–6).

In the last 10–15 years commercial production of primarily ballan wrasse, *Labrus bergylta*, has developed to meet the increased demand and to address sustainability and biosecurity issues related to using wild-caught wrasse. In recent years, considerable improvements with regards to delousing efficiency, disease management and welfare of cleaner fish have been made (4, 7). However, there are still large knowledge gaps regarding how common operational procedures affect the welfare of ballan wrasse, emphasized by reports of high losses during production and when used in sea cages (8–10). Transport of cleaner fish, by boat and/or vehicle, is an essential procedure of the production process and often occurs over long distances. Most of the previous studies on welfare issues related to transport of cleaner fish have focused on lumpfish when transported from land-based hatcheries to co-stocking with salmon in sea-cages (8, 9). In ballan wrasse production, transport between land-based hatcheries and on-growing facilities is common, and episodes of increased mortality have been reported after such transport.

Generally, the transport of live fish is one of the most critical operations in aquaculture, in which fish are exposed to multiple handling events and large variations in environmental factors within a short period of time. These are events that can initiate a severe stress response in farmed fish. In response to stress cortisol, the main stress hormone in teleost fishes, is released (11, 12) enabling stress coping mechanisms by making the resources needed to cope with the stressful stimuli available. Energy required for stress coping is redistributed from maintenance functions such as growth and immune reactions (12). In this vein, the sum of the high intense stressor of transport together with the post transport low intensity stressor have been suggested to affect post transport survival (13). Accordingly, stress has been discussed as one of the underlying factors for delayed mortality syndrome or hauling mortality, referring to mortality appearing days or even weeks after transport (13). Factors that have been suggested to affect the post-transport outcome include water quality, confinement, density, holding container design, handling procedures, and agonistic behavior reviewed by Harmon (13–15).

There is a strong relationship between plasma cortisol levels and the release of this hormone from the fish to the water, reviewed by Scott and Ellis (16). Accordingly, the release rate of cortisol to the water has been used as a non-invasive welfare indicator in laboratory studies (17–19) and in commercial aquaculture settings (20). Fish transport in closed tanks in hauling vehicles with or without external water exchange and in well-boats is a common practice in aquaculture. In such closed transports, cortisol will accumulate over time. Therefore, measurements of water cortisol concentrations can be used as an integrative tool for assessing the total stress burden associated with transport. However, its use as a monitoring tool for fish welfare during transport is still very limited.

Using anesthetics prior and during transport is a common practice to lower the metabolism of the fish, thus reducing toxic metabolite production, reviewed in Harmon (13). Anesthetics are also used to lessen the stress response due to handling and transport (13, 21–23). This is achieved by sedating the fish enough to reduce the stress response and the risk of injury, while fish are still able to maintain swimming and an upright position. Therefore the proper dosage is critical and will vary with species and fish size. AQUI-S^®^, an isoeugenol based sedative, has been used before and during transport to reduce transport stress in several fish species (22, 24, 25). It has proven to be a good sedative for different types of handling procedures, since several fish species go directly into the narcatic (resting) state without a pre excitement phase (26). Although AQUI-S^®^ is a widely used anesthetic the mechanisms of action are not clearly understood. Although Aqui-S has been reported to mitigate stress in several fish species, other studies indicate that the stress mitigating properties of AQUI-S^®^ are species specific (25, 26). If sedation with AQUI-S^®^ affects post transport survival and welfare in ballan wrasse is to our best knowledge unknown.

The objective of this study was to investigate the relationship between transport stress and post transport mortality at a commercial Ballan wrasse on-growing facility. The effect of AQUI-S^®^ sedation on transport stress was quantified by cortisol release rate to the water in the transport tanks, and effects of transport stress on post-transport mortality was investigated by relating cortisol release during transport to mortality rates at the on-growing facility.

## 2 Materials and methods

### 2.1 Transports

The study included transports from 2 commercial hatcheries producing ballan wrasse larvae, all transports were delivered to the same receiving land-based farm for further on-growing. Fish were loaded on to transport trucks by lowering the water level in the tank and then vacuum pumping fish into transport tanks. The loading procedure took between 1 and 3 h. The transports were performed in two trucks with 10 or 12 internal tanks, with a volume of 1.2 and 1.0 m^3^, respectively. Oxygen saturation (%), temperature (°C) and pH were monitored and logged in the transport tanks. Transport time, fish weight and density, temperature, pH and oxygen saturation during the transport are presented in Table 1. All fish from the same transport were offloaded into one tank at the receiving farm, in which they remained throughout the 25-day study. Dead fish from each tank were counted and removed daily.

**Table 1:** Conditions during truck transports of juvenile Ballan wrasse between hatcheries and a land based on-growing facility.

	Treatment									
	AQUI-S^®^					No AQUI-S^®^				
Transport time (h)	16.25	19.50	17.25	15.25	16.25	20.75	17.50	18.50	15.25	12.50
Fish weight (g)	2.6	1.7	2	2.2	1.8	1.5	2	1.7	2.2	2.3
Tank volume (m^3^)	1	1	1	1	1	1	1	1	1,2	1
Fish density (kg m^−3^)	29.3	19.8	12.6	19.8	13.4	12.6	19.6	10.8	28.8	21.1
Temp (min-max; °C)	12.1–13.1	11.8–13.1	12.2–13.3	11.8–13.6	11.8–13.3	11.2–12.5	11.4–12.5	11.4–14.0	11.9–13.5	–
pH (min-max)	6.9–7.6	6.9–7.7	6.7–8.1	7.2–7.4	7.1–7.9	7.3–7.9	7.3–7.5	7.3–7.5	7.2–7.5	–
Oxygen (min–max; %)	107–122	107–132	106–132	103–117	105–138	102–121	106–123	92–128	101–128	85–143

To investigate if AQUI-S reduced stress during transport procedures, AQUI-S was used both during loading and transport during four transports. 0.5 h before loading AQUI-S (2.5 μL/L) was added directly to the fish tanks in the hatchery. In the tanks aboard the hauling vehicle the same dose of AQUI-S was premixed in the tank water before the fish were loaded. Six of the followed transports fish were not treated with AQUI-S^®^, Table 1.

### 2.2 Water sampling and analysis

#### 2.2.1 Water sampling

One liter water samples were collected from the water supply used to fill up transport tanks at the hatchery (basal levels). Post transport water samples (1 L) were randomly collected from four of the tanks on the transport vehicles, directly when fish were offloaded. The water samples were frozen and stored at −20°C until analysis.

#### 2.2.2 Water cortisol analysis

Analysis of accumulated water cortisol during the transport followed a method described by McWhinney et al. (27) with some modifications. Water samples were spiked with 10 ng internal standard (cortisol d4) to correct for matrix effects and for losses in sample extraction, concentration, and analysis. Samples were loaded onto activated Oasis HLB 6 cc (200 mg) solid-phase extraction cartridges (Waters, Milford, MA, United States). After loading 200–300 mL of the samples, the columns were washed with 3 mL milliQ water followed by 3 mL of 20% methanol. Samples were eluted with 5 mL 100% ethyl acetate, dried at 50°C, and reconstituted in 200 μL of 40% methanol with 5 mmol/L ammonium formate and 0.1% formic acid. Separation was achieved on a BEH C8 column (Waters, Milford, MA, United States) using a solvent gradient consisting of 5 mmol/L ammonium formate and 0.1% formic acid in water and methanol. Cortisol content was analyzed with a tandem mass spectrometer (Waters TQ-S, Milford, MA, United States) operated in negative electron spray ionization mode with the following MRM acquisition parameters (precursor and product ions); 407.1 > 331.05, 407.1 > 331.1 for cortisol and 411.1 > 335.05, 411.1 > 335.1 for cortisol d4 (IS).

##### 2.2.2.1 Cortisol release rate

In fish, cortisol in unconjugated form is mainly excreted through the gills to the water and it has previously been shown that free cortisol in the water is directly related to the cortisol concentration in the blood of fish (18). On this basis, the excretion rate of cortisol has been used as a stress indicator in commercial fish farms (20). The excretion rate of cortisol to the water during transport from the hatchery to the receiving on-growing facility, was calculated using the formula:

$\begin{array}{l} \left(\left[{\mathrm{Cortisol}\;\mathrm{transport}}_{\mathrm{end}}\right]-\left[{\mathrm{Cortisol}\;\mathrm{transport}}_{\mathrm{start}}\right]\right)/\\ \left({{\mathrm{biomass}}_{\mathrm{tank}}}^{*}\;\mathrm{transport}\;\mathrm{time}\right) \end{array}$

[Cortisol transport _start_] is the cortisol concentration in the water before fish are loaded. [Cortisol transport _end_] is the cortisol concentration in the tank at the transport end. Biomass _tank_ is the biomass of the fish in the transport tank and transport time is the time between loading and unloading the fish. [cortisol transport _start_] was below the detection limit of the analysis method (0.1 μg L-1) and therefore set to 0.

### 2.3 Statistical analyses

All values are presented as mean ± standard error unless otherwise specified. The effects of AQUI-S^®^ treatment on cortisol excretion rate during transport was investigated by a student’s *T*-test. Effect of transport on mortality during five-day intervals following transport was investigated by a repeated measure analysis of variance (ANOVA). Dunnet’s *post hoc* test was employed to investigate differences in mortality during the first five days interval after transport compared to the following four five-day intervals. Factors affecting accumulated mortality 25 days after transport was investigated with an analysis of covariance (ANCOVA) with transport treatment (AQUI-S^®^ or no AQUI-S^®^ treatment) as categorical factors and cortisol excretion rate during transport and the mean size of the transported fish as continuous predictors. *p* < 0.05 was set as the threshold for statistical significance.

## 3 Results

### 3.1 Water cortisol

**Figure 1:**
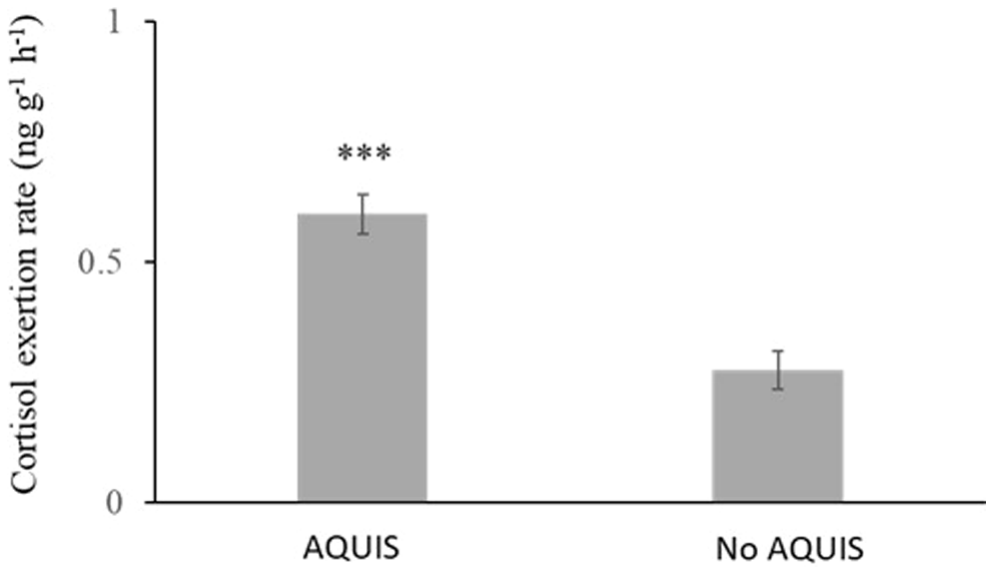
Cortisol excretion rate in Ballan wrasse during truck transportation between hatcheries and an on-growing facility. Fish were treated with AQUI-S during loading and transport in four cases and did not receive AQUI-S treatment before and during the transport in six cases. An asterisk (***) marks a significant difference at the level of *p* < 0.001.

Ballan wrasse sedated with AQUI-S^®^ during loading and transport had a significantly higher cortisol excretion rate compared to fish transported without AQUI-S^®^ treatment (*T*_(8)=_ 5.5, *p* < 0.001) Figure 1.

### 3.2 Mortality after transport

**Figure 2:**
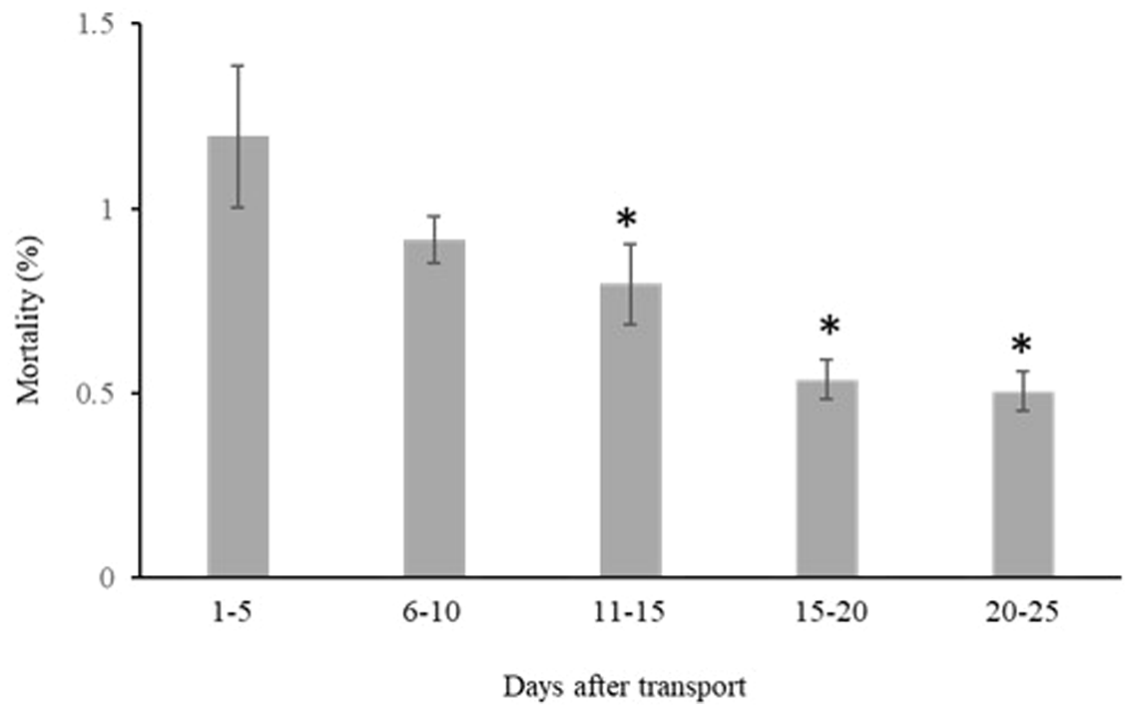
Accumulated mortality of Ballan wrasse in five-day periods post-transport at the receiving on-growing facility. Fish from each transport (10 transports in total) were kept in separate tanks at the receiving on-growing facility for 4 weeks. Mortality was recorded daily. For conditions during transport see Table 1. An asterisk (*) marks a significant difference (*p* < 0.05) versus the first 5-day period after transport.

Mortality changed over time during the first 25 days after transport (Repeated measure ANOVA; *F*_(4, 36)_ = 13, *p* < 0.001), leading to significantly lower values day 11–15, 16–20, and 20–25 compared to mortality during the first five-day period after transport (day 1–5, *p* < 0.01, *p* < 0.001 and p < 0.001 respectively), Figure 2. There was no significant difference in mortality between the first (day 1–5) and the second (day 6–10) period (*p* < 0.067).

**Figure 3:**
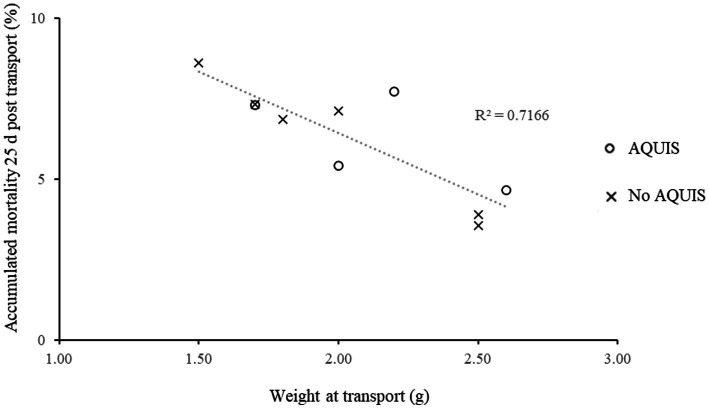
Relationship between the weight of Ballan wrasse juveniles at transport and mortality 25 days post-transport, when transported from hatcheries to a land based on-growing facility. The fish from each transport were kept in separate tanks at the on-growing facility. Mortality was recorded daily.

The results from the ANCOVA showed a significant relationship between fish weight at transport and accumulated mortality 25 days after transport (*F*_(1, 6)_ = 17, *p* < 0.01), Figure 3. However, there were no significant effects of AQUI-S treatment (*F*_(1, 6)_ = 0.0001, *p* < 0.98) on accumulated mortality 25 days after transport. Neither, was there a significant relationship between cortisol excretion rate during transport and accumulated mortality 25 days after transport (*F*_(1, 6)_ = 0.18, *p* < 0.98).

## 4 Discussion

In general transport is one of the most stressful and critical operations in fish farming, including handling, crowding, pumping and potential sub-optimal water quality, and in addition motion in the transport vessel (13, 23). In this study, the average cortisol excretion rate to the water in the transport tanks ranged between 0.15 and 0.6 ng g^−1^ h^−1^. Laboratory studies with rainbow trout and salmon show that cortisol excretion rate can increase from 0.01 to values up to 0.6–7 ng g^−1^ h^−1^ during stressful events, reviewed in Scott and Ellis (18). The initial process of loading to the transport tanks requires several novel stressors within a short time period, i.e., handling, pumping and a new environment, whereas the long transport time may act as a recovery period if water quality is maintained. The average cortisol extraction rate to the water in the transport tanks can be viewed as the total stress burden during transport. Considering this, and that the cortisol release rate to the transport tanks in this study was just under the values in Scott and Ellis (18), the results might reflect that fish are recovering from an acute-intense stress that is associated with additive stressors during the loading process. However, when comparing cortisol excretion rates, it is important to note that higher baseline levels of plasma cortisol have been reported in ballan wrasse compared to rainbow trout however both species seem to have similar cortisol levels in response to acute intense stress (28). This emphasizes that further studies on stress responsiveness, and the relationship between plasma cortisol and cortisol release rate to the water, are needed to verify the impact of potential stressors on the release rate of cortisol in ballan wrasse.

In this study light sedation with AQUI-S^®^ during loading and transport increased cortisol excretion rate. Similar results have bene reported in Gilthead seabream, showing that AQUI-S^®^ sedation elevated plasma cortisol levels after 6 h simulated transport (25). In a comparable study with Gilthead seabream clove oil, an another isoeugenol based sedative, also increased plasma cortisol levels after transport (29). Based on the elevated post transport plasma cortisol values together with differences in gene expression in the head kidney, the authors suggest that clove oil prolongs the time needed for stress recovery (29). As mentioned above, it is possible that the relatively low cortisol release rate during transport in our study reflects that fish were able recover from the initial intense stress associated with loading during the long transport. Also in line with (28), our findings suggest that AQUI-S^®^ sedated fish take longer to recover and habituate to transport tanks thus have a higher overall cortisol release rate. However, these results are in contrast to studies in salmon that show that AQUI-S^®^ has stress reducing capabilities (22, 30). In Atlantic salmon smolts, sedation just before onloading to the transport truck and again 15 min before offloading resulted in lower post-transport plasma cortisol levels (22). Still, it is important to note that in the studies (22, 30) that demonstrate that AQUI-S^®^ has stress reducing effects during transport procedures, AQUI-S^®^ was not used the whole transport and fish were allowed to recover in water without AQUI-S^®^ before sampled. Thus, it cannot be excluded that the sedation protocol for reducing transport stress in Atlantic salmon smolts (22) might also have stress reducing effects in Ballan wrasse, if only used during handling and onloading operations and not during transport.

In line with the suppressive effects of AQUI-S^®^ on the cortisol response in Atlantic salmon during transport procedures it has also been demonstrated that channel catfish (*Ictalurus punctatus*) exposed to confinement and hypoxia when AQUI-S^®^ sedated have a reduced cortisol response (31). There are also other studies that report no effect of AQUI-S^®^ on the plasma cortisol response such as when used for crowding rainbow trout (*Oncorhynchus mykiss*) (32) or striped bass exposed to low water levels (33). In addition to the mentioned effects of AQUI-S^®^ on cortisol dynamics during stress recovery, species specific effects of the sedative on the stress response might also contribute to discrepancies between studies (26, 34).

Generally, light sedation is considered to mitigate stress related mortality in transported fish reviewed by Harmon (13). Iversen and Eliassen (22) reported that beside the stress reducing effects of AQUI-S^®^ it also resulted in higher post transport survival rates in Atlantic salmon smolts. In our study we could not detect any effects of AQUI-S^®^ on post transport mortality. Interestingly, size at transport showed a close negative relationship with accumulated mortality 25 days after transport. Furthermore, in our study, mortality was highest (~1%) the first 5 days after transport to the on-growing facility. After 5 days mortality decreased and 25 days post transport the accumulated mortality was ~4%. In one of the few other studies performed on ballan wrasse transport, Jonassen and Foss (35) showed similar mortality values after transporting larger ballan wrasse from a land-based facility to open sea- cages (by truck and/or well-boat). In that study the cumulative mortality in the sea-cages was 1.1 to 1.3% seven days after transfer and between 4.4 and 5.8% 1 month post -transport. However, in the mentioned study, it is pointed out that the large difference in rearing conditions between the land and sea site and the increased environmental variability at sea has a greater impact on survival than the transport stress. In the present study, fish were reared in similar conditions at the hatchery and at the on-growing facility. Thus, the observed post transport mortality is likely linked to transport conditions and size of the fish. However, it is important to note that data on size related mortality in non-transported fish is needed to verify the actual impact of transport on mortality in farmed ballan wrasse. Still, the strong relationship between fish weight at transport and post-transport mortality in the present studym suggests that farmers should include size at transport as an important factor to consider during planning and establishing best practice protocols for juvenile ballan wrasse transports.

In conclusion, this study shows that slight sedation with AQUI-S^®^ neither had a mitigating effect on cortisol release rate during transport nor reduces post-transport mortality in ballan wrasse. Moreover, somewhat in contrast to what was expected, AQUI-S^®^ treatment during loading and transport increased cortisol excretion rate to the water in the transport tanks, suggesting a stimulating effect of AQUI-S^®^ on the stress axis in ballan wrasse. Furthermore, the cortisol release rate in the present study was rather low in comparison with stressed salmonids. This suggests a species difference in cortisol release rate and/or that during the long transport the ballan wrasse have been recovering from the initial stressful phase of handling and onloading to the transport tanks. The latter is supported by the stimulating effect of AQUI-S^®^ on the cortisol response observed in our study and other studies (25, 29) indicating that using AQUI-S^®^ during transports prolongs the time needed for stress recovery. Overall, this study demonstrates that water cortisol can be used as an integrative tool to assess stress during transport. Still, studies linking cortisol release rate during transport to negative welfare and health effects, and ultimately leading to delayed mortality syndrome or hauling mortality, are required for implying negative transport consequences. Moreover, considering that the negative impact of stress on animal welfare is related to the intensity, duration, and frequency of the stressors (36), further studies linking the dynamics of cortisol release rate to fish health and welfare is needed.

## Data availability statement

The raw data supporting the conclusions of this article will be made available by the authors, without undue reservation.

## Ethics statement

Ethical approval was not required for the study involving animals in accordance with the local legislation and institutional requirements because we followed commercial fish transport with normal routines. We used water born cortisol as a stress indicator of transported fish. Thus, fish was not exposed extra stress due to sampling.

## Author contributions

SC: Investigation, Writing – review & editing. TJ: Methodology, Writing – review & editing. ES: Writing – review & editing. HÅ: Investigation, Writing – review & editing. AI: Funding acquisition, Project administration, Writing – review & editing. CS: Investigation, Methodology, Writing – original draft, Writing – review & editing. TW: Methodology, Writing – review & editing. EH: Conceptualization, Formal analysis, Investigation, Methodology, Project administration, Writing – review & editing.
