# Corrigendum: Does sedation with AQUI-S^®^ mitigate transport stress and post transport mortality in ballan wrasse (*Labrus bergyltae*)?

**DOI:** 10.3389/fvets.2024.1386051

**Published:** 2024-03-15

**Authors:** Sara Calabrese, Thor Magne Jonassen, Endre Steigum, Helga Øen Åsnes, Albert Kjartan Dagbjartarson Imsland, Carolina Serra Saude, Truls Wergeland, Erik Höglund

**Affiliations:** ^1^Norwegian Institute for Water Research, Bergen, Norway; ^2^Akvaplan-niva AS, Tromsø, Norway; ^3^Department of Biological Sciences, University of Bergen, Bergen, Norway; ^4^MOWI ASA, Bergen, Norway; ^5^University of Agder, Kristiansand, Norway

**Keywords:** delayed mortality, sedation, transport, Cleanerfish, aquaculture, fish welfare

In the published article, there was an error. There is an error in citing references in the text.

A correction has been made to Discussion, paragraph 2, page 4.

This sentence previously stated:

“Still, it is important to note that in the studies (x,x andx) that demonstrate that AQUI-S^®^ has stress reducing effects during transport procedures, AQUI-S^®^ was not used the whole transport and fish were allowed to recover in water without AQUI-S^®^ before sampled.”

The corrected sentence appears below:

“Still, it is important to note that in the studies (22,30) that demonstrate that AQUI-S^®^ has stress reducing effects during transport procedures, AQUI-S^®^ was not used the whole transport and fish were allowed to recover in water without AQUI-S^®^ before sampled.”

The authors apologize for this error and state that this does not change the scientific conclusions of the article in any way. The original article has been updated.

